# Supplementary material for: Novel Pyrrolidinium-Functionalized Styrene-b-ethylene-b-butylene-b-styrene Copolymer Based Anion Exchange Membrane with Flexible Spacers for Water Electrolysis
Source: Membranes (Basel). 2023 Mar 13;13(3):328. doi: 10.3390/membranes13030328 (PMC10057012; doi:10.3390/membranes13030328)
Supplement: Supplementary file 1 [file membranes-13-00328-s001.zip › membranes-2258556-supplementary.pdf]

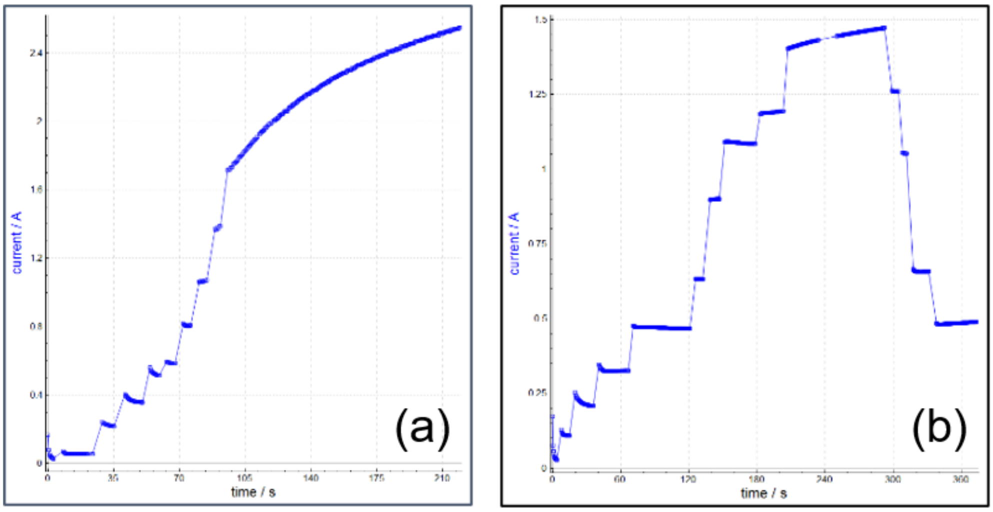

Figure S1. (a) potentiostatic conditioning in 0.1 M KOH electrolyte; (b) potentiostatic conditioning in UPW.
